# Batimastat Induces Cytotoxic and Cytostatic Effects in In Vitro Models of Hematological Tumors

**DOI:** 10.3390/ijms25084554

**Published:** 2024-04-22

**Authors:** Raquel Alves, Ana Pires, Joana Jorge, Joana Balça-Silva, Ana Cristina Gonçalves, Ana Bela Sarmento-Ribeiro

**Affiliations:** 1 Laboratory of Oncobiology and Hematology (LOH), University Clinics of Hematology and Oncology, Faculty of Medicine (FMUC), University of Coimbra, 3000-548 Coimbra, Portugal; raquel.alves@fmed.uc.pt (R.A.); ana.pires@histologix.com (A.P.); jjorge@fmed.uc.pt (J.J.); joana.b.silva@edu.nms.unl.pt (J.B.-S.); absarmento@fmed.uc.pt (A.B.S.-R.); 2 Coimbra Institute for Clinical and Biomedical Research (iCBR), Group of Environmental Genetics of Oncobiology (CIMAGO), FMUC, University of Coimbra, 3000-548 Coimbra, Portugal; 3 Center for Innovative Biomedicine and Biotechnology (CIBB), 3004-504 Coimbra, Portugal; 4 HistologiX, BioCity, Innovation, Pennyfoot St., Nottingham NG1 1GF, UK; 5 NOVA Medical School, New University of Lisbon, 1150-090 Lisbon, Portugal; 6 Hematology Service, Centro Hospitalar Universitário de Coimbra, Unidade Local de Saúde de Coimbra, 3000-061 Coimbra, Portugal

**Keywords:** matrix metalloproteinase, batimastat (BB-94), acute leukemia, multiple myeloma, myelodysplasia, apoptosis

## Abstract

The role of metalloproteinases (MMPs) in hematological malignancies, like acute myeloid leukemia (AML), myelodysplastic neoplasms (MDS), and multiple myeloma (MM), is well-documented, and these pathologies remain with poor outcomes despite treatment advancements. In this study, we investigated the effects of batimastat (BB-94), an MMP inhibitor (MMPi), in single-administration and daily administration schemes in AML, MDS, and MM cell lines. We used four hematologic neoplasia cell lines: the HL-60 and NB-4 cells as AML models, the F36-P cells as an MDS model, and the H929 cells as a model of MM. We also tested batimastat toxicity in a normal human lymphocyte cell line (IMC cells). BB-94 decreases cell viability and density in a dose-, time-, administration-scheme-, and cell-line-dependent manner, with the AML cells displaying higher responses. The efficacy in inducing apoptosis and cell cycle arrests is dependent on the cell line (higher effects in AML cells), especially with lower daily doses, which may mitigate treatment toxicity. Furthermore, BB-94 activated apoptosis via caspases and ERK1/2 pathways. These findings highlight batimastat’s therapeutic potential in hematological malignancies, with daily dosing emerging as a strategy to minimize adverse effects.

## 1. Introduction

Tumor cells are highly influenced by the surrounding microenvironment, composed of cellular and molecular components, to provide signals that promote tumor progression, favor apoptosis escape, and might impact drug response levels [1]. In the hematopoietic tissue, the bone marrow microenvironment (BMM) is tightly controlled to promote the proper proliferation and differentiation of blood components [2]. Deregulation of BMM homeostasis has been associated with matrix metalloproteinase (MMP) activity [2,3]. MMPs belong to a family of zinc-dependent endopeptidases that can degrade the majority of components in the extracellular matrix (ECM) [4]. In addition to their role in ECM remodeling, MMPs have been described as important players in tumorigenesis due to the modifications of several signaling pathways by influencing the bioavailability and regulation of cytokines, growth factors, and receptors [5,6].

The activity of these endopeptidases has been associated with the development of hematological malignancies, including acute myeloid leukemia (AML), myelodysplastic neoplasms (MDSs), and multiple myeloma (MM) [7,8,9]. AML has its origin in the hematopoietic stem and/or progenitor cells and constitutes a heterogeneous disorder. This neoplasia is characterized by a block in the normal development of myeloid cells and increased cell proliferation, causing immature myeloid cells to accumulate in the bone marrow, peripheral blood, and other tissues [10]. MDSs are clonal neoplasms of hematopoietic stem cells characterized by cytopenias and morphologic dysplasia, having a high probability of progressing to AML [11]. MM is a hematological malignancy characterized by the presence of abnormal clonal plasma cells in the bone marrow producing clonal immunoglobulins and by the presence of defining myeloma events [12]. Despite the evolution in treatment options for these neoplasias, they continue to be associated with poor outcomes and high relapse rates.

The contribution of MMPs to cancer development led to the exploration of these endopeptidases as therapeutic targets in different neoplasias. Over the years, several MMP inhibitors (MMPis) have been evaluated in in vitro models and clinical trials, as in the case of batimastat and marimastat [13]. MMPis are divided into four categories: collagen peptidomimetic, non-peptidomimetic, tetracycline derivatives, and bisphosphonates [14]. Batimastat (BB-94) is a low-molecular-weight inhibitor and belongs to the collagen peptidomimetic category. As a broad-spectrum MMP inhibitor, BB-94 was the first inhibitor to be tested in different models, such as cell lines and metastatic models, as well as in cancer patients [13]. The action of this inhibitor has been associated with cytostatic effects and interfering with signaling pathways, such as the MAPK and PI3K pathways [5]. However, the inhibition of MMP has been poorly explored in hematological malignancies despite the critical role of the microenvironment in hematopoietic tissues.

In this study, we investigated the effects of batimastat in single-administration and daily administration schemes in AML, MDS, and MM cell lines. We highlight the efficacy of BB-94 inducing apoptosis and cell cycle arrests in hematological models, particularly the impact of daily administration of lower doses to reduce treatment toxicity.

## 2. Results

### 2.1. Hematological Neoplasia Cell Lines Express Different Levels of MMPs

**Figure 1:**
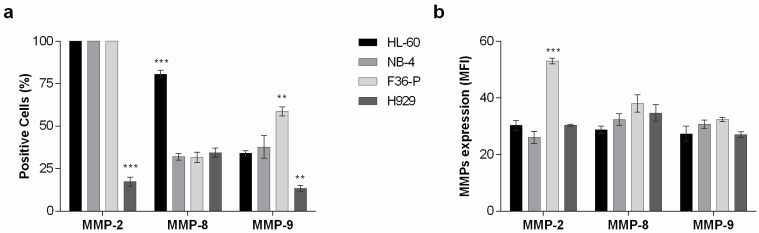
MMPs expression levels in HL-60, NB-4, F36-P, and H929 cell lines. (**a**) The percentage of cells that express MMP-2, MMP-8, and MMP-9, and (**b**) the expression levels represented by mean fluorescence intensity (MFI). Results represent the mean ± SEM obtained from 3 independent experiments. ** *p* < 0.01; *** *p* < 0.001 (comparisons between each other).

Since batimastat is an MMP inhibitor and the action of MMP-2, MMP-8, and MMP-9 has been associated with hematopoietic tissue [7,15,16], we first investigated the expression of these enzymes in our models (Figure 1). MMP-2 was expressed in all HL-60, NB-4, and F36-P cells, while only 17.3 ± 2.6% of H929 cells expressed this MMP (Figure 1a). In terms of the amount of MMP-2 in each cell (represented by the MFI), this expression was significantly higher in F36P cells (*p* < 0.001) compared with other cell lines that reveal similar expression of MMP-2 (Figure 1b). For MMP-8, 80.7 ± 2.2% of HL-60 cells expressed this protein, being significantly higher (*p* < 0.001) compared to the 30.2 ±2.0% of MMP-8 positive cells in NB-4, the 31.7 ± 3.0% in F36-P, and 34.3 ± 2.6% in H929 models (Figure 1a). Despite these differences, no significant alterations were observed between the four cell lines regarding the amount of MMP-8 per cell (Figure 1b). The percentage of positive cells for MMP-9 was more heterogeneous among the four cell lines but without differences in the expression levels (Figure 1). H929 cells presented a lower percentage of positive cells for MMP-9 (13.3 ± 1.7%; *p* < 0.01) when compared to HL-60 (34.0 ± 1.5%), NB-4 cells (37.7 ± 6.7%), and F36-P cells (58.7 ± 2.6%; *p* < 0.01) (Figure 1a).

### 2.2. Batimastat Reduces Cell Viability and Cell Density in Hematological Neoplasia Cell Models

**Figure 2:**
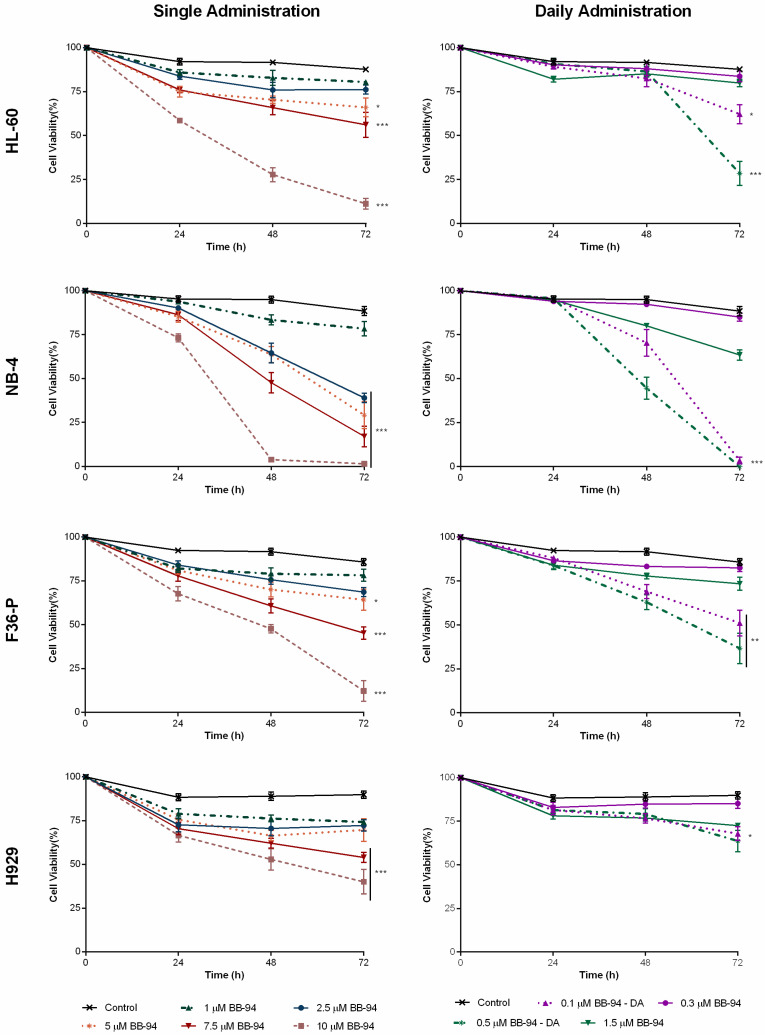
Dose–response curves of batimastat (BB-94) in single-administration and daily administration schedules for the four hematological cell lines. Cells were incubated in the absence and presence of different concentrations of BB-94 during 72 h with different administration schemes. Results were expressed in percentage (%) and represent the mean ± SEM obtained from 4 independent experiments. * *p* < 0.05; ** *p* < 0.01; *** *p* < 0.001 (comparison with control).

We evaluated the impact of BB-94 on cell viability (Figure 2) and cell density (Supplementary Figure S1) on our cell models based on single and daily administration schedules. BB-94 decreases cell viability and density in a dose-, time-, administration-scheme-, and cell-line-dependent manner. In a single-administration scheme, the most sensitive model to BB-94 was the NB-4 cell line, followed by HL-60 and F36-P cells, and the least responsive were the H929 cells. For example, after 48 h of drug exposure, for the highest concentration tested (10 µM), the cell viability was approximately 3.9 ± 1.3% in NB-4 cells versus 53 ± 6.0% in H929 cell lines (Figure 2). The mathematical BB-94 half-maximal inhibitory concentration (IC50) was 7.9 ± 1.6 µM for NB-4 cells, 9.8 ± 3.9 µM in HL-60 cell line, 12.1 ± 1.2 µM in F36-P cells, and 18.0 ± 1.6 µM in the MM model (H929 cells). We further investigated the influence of lower doses administrated daily (DA) compared to total concentration administrated in a single time. In all cell lines, a significant reduction in cell viability was observed with DA compared to the full dose at one point. Again, the most pronounced effect was observed in NB-4. In this cell line, the single administration of 1.5 µM of BB-94 reduced approximately 20% of the cell viability, while DA of 0.5 µM of the drug killed almost all the cells after 72 h of treatment (*p* < 0.001). Despite some statistical differences between 0.1 µM DA of BB-94 and 0.3 µM of BB-94 (*p* < 0.001), the H929 cell line was the model where the administration scheme showed a lower impact on cell viability. When compared with single-administration data, 0.5 µM of BB-94 administered daily (totaling 1.5 µM of BB-94) was able to induce higher effects than 7.5 µM of the drug at 72 h in HL-60, NB-4, and F36P cells. At 72 h, with a five times lower drug exposure, the viability reduced to 28.4 ± 6.8% and 36.6 ± 8.6% with 0.5 µM of BB-94 in DA in the HL-60 and F36-P cells, respectively, while with 7.5 µM in a single administration, the viability only reached 56.1 ± 7.2% and 45.2 ± 3.6% in the same cells.

The cytostatic effect of BB-94 was observed in all cell lines (Supplementary Figure S1), being more pronounced in the acute myeloid leukemia models. Similar to what was observed in cell viability, the daily administration scheme also presented a higher effect in reducing the cell density compared to the single-dose administration. The H929 cells were the exception, where the DA of BB-94 did not reach statistical significance.

In the non-tumoral cell model, i.e., the IMC cells, BB-94 did not show an impact on cell viability and only induced a small reduction in cell density (Supplementary Figure S2).

### 2.3. Batimastat Triggers Apoptosis as Mechanism of Cell Death

**Figure 3:**
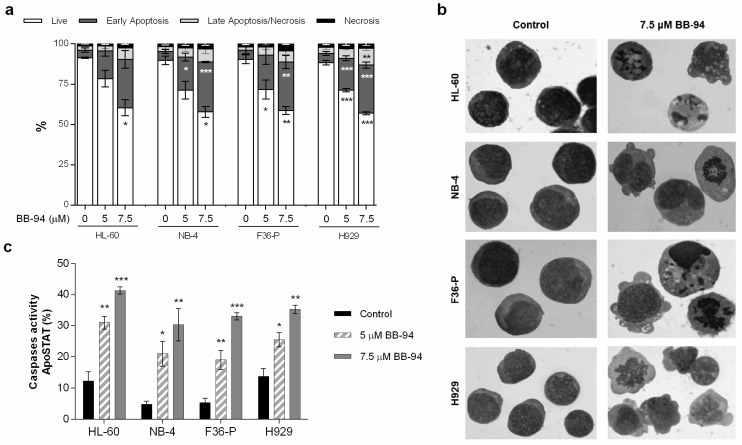
Analysis of cell death induced by batimastat in HL-60, NB-4, F36-P, and H929 cell lines. (**a**) The type of cell death was identified by annexin V/propidium iodide staining and analyzed by flow cytometry; data were expressed as the percentages (%) of live, early apoptotic, late apoptotic/necrotic, and necrotic cells. In (**b**), cell morphology was analyzed in smears stained with May–Grünwald–Giemsa staining (amplification: 500×). (**c**) The caspase activity was determined by flow cytometry using ApoSTAT probe as a marker of apoptosis. Data were expressed as the percentage (%) of positive cells for ApoSTAT labeling. Results were obtained after 48 h of incubation and represent mean ± SEM of at least 3 independent experiments. * *p* < 0.05; ** *p* < 0.01; *** *p* < 0.001 (comparison with control).

To further analyze the mechanism of cell death induced by BB-94, we selected the time point of 48 h and the doses of 5 and 7.5 µM of the MMP inhibitor. In flow cytometry analysis, using the AV/PI staining, we observed a significant reduction in viable cells associated with an increase in cells in the early stages of apoptosis and in late apoptosis/necrosis (Figure 3a). BB-94 did not induce alterations in the percentage of necrotic cells. For example, in F36-P cells for the tested conditions, a dose-dependent reduction in cell viability was observed. In cells treated with 7.5 µM of BB-94, the viable cells were 58.7 ± 2.4% compared to 90.3 ± 2.7% of viable cells in the control condition (*p* < 0.01). This is accompanied by an increase to 30.0 ± 4.0% of cells in early apoptosis versus the 5.6 ± 2.3% observed in the untreated condition (*p* < 0.01).

Corroborating these findings, the morphological evaluation showed typical features of apoptosis in all cell lines exposed to BB-94, as observed in Figure 3b. The drug caused the blebbing of cell membranes, chromatin condensation, and nuclear fragmentation. Furthermore, the apoptosis triggered by BB-94 was associated with a statistically significant increase in caspase activity (Figure 3c) that is dose-dependent. These results are represented by the increase in positive cells for ApoSTAT labeling (Figure 3c). We also observed an increase in caspase expression levels (MFI; Supplementary Figure S3). For instance, in HL-60 cells, the highest concentration of BB-94 increased the percentage of caspase-positive cells by approximately 30% compared to the control (*p* < 0.001), which is associated with almost three times more caspases per cell (*p* < 0.001).

### 2.4. Batimastat Induces Cell Cycle Arrest in a Cell-Line-Dependent Manner

To understand if BB-94 cytostatic effects were due to the cell cycle arrest, we analyzed cell cycle progression by flow cytometry. We observed that the BB-94 effect on cell cycle distribution was dependent on the cell line (Table 1, Supplementary Figure S4).

In HL-60 cells was observed a cycle arrest in the G0/G1 phase, with a significant increase in cells in this phase (53 ± 0.6%) in the conditions of 7.5 µM of BB-94 compared to 43.0 ± 1.0% observed in the control condition (*p* < 0.001). A significant cytostatic effect was also observed in the NB-4 cell line, but the arrest occurred in the S phase. For this cell line, we observed a significant increase in % of cells in this cell cycle phase that is independent of the dose of BB-94, with 51.3 ± 0.9% of cells in the S phase in 7.5 µM BB-94 treatment versus 36.7 ± 1.8% in untreated cells. In F36-P and H929 cells, there was a slight increase in cells in the G2/M and G0/G1 phases, respectively, without statistical significance (Table 1). With the same technique, it was possible to evaluate the DNA fragmentation typical of apoptotic cells, which corresponds to cells in the sub-G1 population (Table 1). In all cell lines, a dose-dependent increase in the sub-G1 population was observed, confirming the previous results that identify apoptosis as the main mechanism of cell death induced by BB-94.

### 2.5. Batimastat Influences Signaling Pathways and MMP Activity

**Figure 4:**
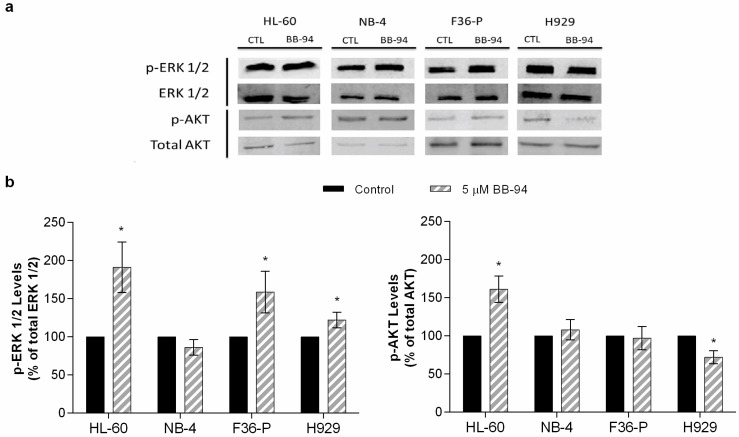
The effects of BB-94 on p-ERK1/2 and p-AKT expression after 48 h of incubation. (**a**) A representative immunoblot of the total and phosphorylated forms of ERK1/2 and AKT in each cell line. (**b**) In untreated and 5 µM of BB-94-treated cells, the phosphorylated ERK 1/2 and AKT levels were quantified and compared with the control. The loading control was performed with an antibody for total ERK1/2 and total AKT, respectively. Data are expressed in percentage (%) as mean ± SEM of 3 independent experiments. * *p* < 0.05 (comparison with control).

The action of BB-94 can be linked with MAPK/ERK and PI3K/AKT pathways activation. To assess the activation of these pathways, we evaluated in all cell lines the phosphorylation levels of ERK and AKT proteins in untreated and treated conditions (Figure 4). The exposure to 5 µM of BB-94 leads to a significant increase in ERK1/2 phosphorylation in HL-60, F36P, and H929 cells. In opposition, in NB-4 cells, only a slight decrease in the activation of this pathway was observed (Figure 4b). In Figure 4b, the AKT activation is demonstrated in HL-60 cells with a significant increase in p-AKT form, while in the H929 cell line, lower levels of AKT phosphorylation are detected in the BB-94-treated conditions. In the remaining cell lines, no alterations were registered in this pathway upon BB-94 exposure.

Complementarily, and since MMP-2 and MMP-9 belong to the gelatinase subgroup, we performed a gelatin zymography assay to measure the activity of these MMPs in the most sensitive cells to BB-94 (Supplementary Figure S5). As observed in Supplementary Figure S4, the BB-94 induced a very small reduction in MMP-2 and MMP-9 activity in NB-4 cells, not reaching statistical differences.

## 3. Discussion

The intricate network between MMPs, BMM remodeling, and hematological malignancies leads to the exploitation of MMP inhibitors as a potential target therapy in these malignant diseases. In this study, we evaluated the therapeutic potential of batimastat (BB-94), an MMP inhibitor, in AML, MDS, and MM in vitro models. Our results demonstrated that the MM cell line was the least sensitive to BB-94, while the AML cells were the most sensitive to this inhibitor. Even among the AML models, a more pronounced effect was observed in the acute promyelocytic leukemia cells. Furthermore, the daily administration schedule with lower doses of BB-94 revealed promising results. In terms of mechanism of action, this inhibitor promoted the activation of apoptosis with the contribution of caspases and ERK1/2 to mediate its cytotoxic effect. A cytostatic effect was also observed and dependent on the cell line.

The robust evidence associating MMPs and tumorigenesis fueled the development of MMP inhibitors and their assessment in clinical trials for various cancer types since the 1990s [13]. In the first clinical trial, batimastat was able to control neoplastic ascites [17,18]. This inhibitor, with its broad-spectrum activity, can, “in theory”, inhibit all MMPs, plus the inhibition of ADAM family members [19]. Despite the promising pre-clinical results, the clinical trials reveal toxic effects that compromise BB-94 efficacy and applicability. However, most of the studies were focused on solid tumors, with a special emphasis on cellular migration, and the clinical trials only included advanced cancer patients [20]. The impact of this inhibitor on hematological malignancies is poorly explored despite the crucial role of MMPs in these diseases.

Our results showed that batimastat’s impact on cell viability and density was cell-line dependent, and this may reflect the differences in MMP expression across the hematopoietic cells. Over the four models studied, the myeloid cells were more sensitive than the lymphoid H929 cells. This could be justified by the higher expression levels of MMP-2 in all myeloid cells versus the lower expression (only 17% of cells) detected in the H929 cell. Corroborating our observations, MMP-2 is mostly produced by multiple myeloma patients’ bone marrow stroma cells rather than the malignant plasma cells [4]. Focusing on AML models, the highest sensitivity of NB-94 to BB-94’s action might be linked with t(15;17), which originates from the PML-RARa fusion gene. Pirillo and colleagues reported that compared with other AML subtypes, the PML-RARa samples presented higher MMP-2 gene expression levels, and this could be associated with EMT characteristics [8,21].

Previous work from our groups reveals that daily administration of lower doses of compounds potentiates their efficacy [22]. In the case of BB-94, a daily administration scheme led to a significant reduction in cell viability in myeloid cells, with better results than five times higher doses in a single administration. This strategy may help to reduce the dose-related side effects already described for BB-94 in other neoplasias.

In accordance with previous studies [23,24,25], BB-94 induced a significant cytotoxic effect mediated by apoptosis with caspase activation in our models. Furthermore, the activation of ERK1/2 in HL-60, F36-P, and H929 cells might be associated with apoptosis activation. The anti-survival role of ERK1/2 has been described in multiple conditions and as a consequence of different stimuli, such as in the case of DNA damage [26]. ERK1/2 can promote the release of cytochrome c in the intrinsic apoptotic pathway or influence the expression levels of cell death receptors from the extrinsic apoptotic pathway [26]. Moreover, ERK may also lead to apoptosis by suppressing survival pathways as AKT signaling [27]. In the H929 cell line, we observed an activation of ERK1/2 associated with a decrease in the AKT pathway, which supports the activation of apoptosis. In HL-60 cells, the AKT activation might be a mechanism of the cells to counteract the BB-94 action [28].

Batimastat inhibits MMPs by binding to their catalytic domain and preventing cleavage of substrate molecules [5]. However, it is important to note that the inhibition of MMP activity can indirectly affect MMP expression levels. When MMP activity is inhibited by compounds like batimastat, it can lead to a feedback mechanism in which cells respond by altering the expression of MMP genes [29]. In our experiments, we did not observe a significant reduction in MMP-2 and MMP-9 activity upon BB-94 exposure, so it is not expected to increase MMP expression, suggesting an off-target effect. Further studies are needed to fully understand the batimastat mechanism of action in hematological malignancies, particularly the role of the AKT signaling pathway.

Complementing apoptosis activation, we also observed cytostatic effects in our cells. As described in the literature, BB-94 induced a cell cycle arrest at the G0/G1 phase in HL-60 cells [23,24,25]. In NB-4 cells, an S-phase arrest was observed, and this difference could be linked with the PML-RARa fusion gene. This fact can be attributed to the impact of the PML-RARA protein on the repair of DNA damage by disrupting the formation and function of PML nuclear bodies (PML-NBs). These PML-NBs play a crucial role in various cellular processes, including DNA repair [30], and the disruption of DNA repair may lead to S-phase arrest.

The large number of BB-94 targets may be beneficial since multiple MMPs have been implicated in the same neoplasia, but at the same time, this leads to higher toxicity and an increase in the frequency of adverse effects. The knowledge obtained from previous studies paves the way for introducing improvements in the MMP inhibitors area. Multiple strategies can be adopted to improve MMP inhibitor efficacy and to decrease toxicity, namely adapting the target site from the catalytic domain to exosites resulting in higher selectivity, modulating the natural MMP inhibitors with TIMP analogs, or improving delivery and targeting strategies with nanoparticles, allowing less toxicity to surrounding tissues [13,19]. All of this, conjugated with clinical trials that included early-stage patients, might reveal the potential of MMP inhibitors as therapeutic strategies. Based on our results, exploring a daily administration scheme of BB-94 complemented with an improved drug delivery system might be an attractive therapeutic option for hematological malignancies.

## 4. Materials and Methods

### 4.1. Cell Culture

We used four hematologic neoplasia cell lines: HL-60, an acute myeloid leukemia model, obtained from the American Type Cell Collection; NB-4, an acute promyelocytic leukemia cell line and H929, a model of multiple myeloma, both obtained from the German Collection of Microorganisms and Cell Cultures (DSMZ); and a myelodysplastic neoplasia model, the F36-P cells, obtained from the European Collection of Cell Cultures. An immortalized normal human lymphocyte cell line (IMC) was used and established in the Cytogenetics and Genomics Laboratory, Faculty of Medicine, University of Coimbra. Cells were cultured in RPMI-1640 medium (Gibco, Invitrogen, Waltham, MA, USA) supplemented with 10% (HL-60 and NB-4) or 20% (F36-P, H929, and IMC) of heat-inactivated fetal bovine serum (FBS; Gibco, Invitrogen), 2 mM glutamine, 100 µg/mL streptomycin, and 100 U/mL penicillin (Gibco, Invitrogen). Cells were grown at initial densities of 0.3 (HL-60 and NB-4) or 0.5 (F36-P, H929, and IMC) × 10^6^ cells/mL and maintained at 37 °C in a humidified atmosphere containing 5% CO_2_.

Batimastat (BB-94; Tocris Bioscience, Bristol, UK) was dissolved in dimethyl sulfoxide (DMSO; Sigma-Aldrich, St. Louis, MO, USA), and aliquots were frozen at −20 °C. Cell lines were incubated in the absence or presence of increasing concentrations of BB-94, ranging from 1 to 10 µM, for 72 h. Additionally, a daily dose administration schedule was performed with 0.1 and 0.5 µM of BB-94, compared with 0.3 and 1.5 µM of BB-94 in single-dose administration in all cell models.

### 4.2. MMP Protein Expression Evaluation

The expression of MMP-2, -8, and -9 was determined by flow cytometry with specific monoclonal antibodies conjugated with fluorescent probes. For MMP-2 and MMP-9, we used antibodies anti-hMMP-2-FITC and anti-hMMP-9-PE (R&D Systems, Minneapolis, MN, EUA), while for MMP-8, we used anti-hMMP-8 (R&D Systems) with a secondary antibody conjugated with APC (Santa-Cruz Biotechnology, Dallas, TX, EUA). For each cell line, 0.5 × 10^6^ cells were collected and incubated with 1 µg of each antibody, according to manufacturer instructions. After staining, cells were analyzed using a six-parameter, four-color FACSCalibur flow cytometer (Becton Dickinson, Franklin Lakes, NJ, USA). At least 10,000 events were acquired using CellQuest software v.3.3 (Becton Dickinson) and analyzed using Paint-a-Gate software v.3.0 (Becton Dickinson). The results are expressed as the percentage of cells positive for each molecule (%) and as mean fluorescence intensity (MFI, arbitrary units), representing the mean ± SEM of 3 independent experiments.

### 4.3. Cell Viability and Proliferation Assessment

The density and viability of the different cell models were assessed every 24 h during 72 h by trypan blue exclusion assay. Briefly, this assay identifies the viable cells by their ability to exclude dye, while dye-stained cells correspond to nonviable cells. Succinctly, cell suspension and trypan blue (Sigma-Aldrich) were combined in equal volumes, loaded into a Neubauer chamber, and counted with an optical microscope. Cell density was determined by the number of viable cells per mL, and cell viability was calculated as the percentage of viable cells in the sample. The results represent the mean ± SEM of four independent experiments.

### 4.4. Cell Death Analysis

Cell death was evaluated by optical microscopy, through morphological assessment of May–Grünwald–Giemsa-stained slides, and by flow cytometry, using the Annexin V (AV) and propidium iodide (PI) double staining. For the following analysis, the different cell lines were incubated with 5 µM and 7.5 µM of BB-94 for 48 h. For morphological studies, the cells were smeared in glass slides and later stained with the May–Gründwald–Giemsa protocol [31]. Briefly, 1 × 10^6^ cells of each study condition were collected and seeded in glass slides. Then, smears were stained for 3 min with May–Grünwald solution (Sigma-Aldrich) and afterward with Giemsa solution (Sigma-Aldrich) for 15 min. Cell morphology was analyzed by light microscopy using a Zeiss Axioskop 2 equipped with a Zeiss Axiocam ICc3.

After 48 h of incubation in the presence of batimastat, the cells were washed with PBS by centrifugation at 500× *g* for 5 min. After that, the cells were stained with annexin V (AV) and propidium iodide (PI) and analyzed as described in Alves et al. [32]. Shortly, 0.5 × 10^6^ cells were collected and washed with PBS, centrifuged at 500× *g* for 5 min, resuspended in 100 μL of binding buffer, and incubated with 5 μL of AV-APC (BD Biosciences, Franklin Lakes, NJ, USA) and 2 μL of PI (BioLegend, San Diego, CA, USA) for 15 min in the dark at room temperature. Cells were then diluted in 300 µL of binding buffer, and the analysis was performed in a FACSCalibur (Becton Dickinson) flow cytometer. At least 10,000 events were acquired using CellQuest software (Becton Dickinson) and analyzed using Paint-a-Gate (Becton Dickinson). Results represent the percentage of each cell population: live cells (AV-/7-AAD-), early apoptotic cells (AV+/7-AAD-), late apoptotic/necrotic cells (AV+/7-AAD+), and necrotic cells (AV-/7-AAD+); and represent the mean ± SEM of at least 3 independent experiments.

### 4.5. Caspase Activity Evaluation

Caspase assessment was performed using the ApoStat Apoptosis detection kit (R&D Systems), according to manufacturer instructions. For that purpose, the cells were incubated for 48 h in the absence or presence of 5 µM and 7.5 µM of BB-94. Afterward, cells were resuspended in 1 mL of PBS and incubated with 1 μg of ApopStat at 37 °C for 15 min. Then, cells were washed and resuspended in 400 μL of PBS for flow cytometer analysis. The results are presented as mean intensity fluorescence (MIF) arbitrary units and as a percentage of cells with activated caspases. The results were expressed as the mean ± SEM of at least 3 independent experiments.

### 4.6. Cell Cycle Analysis

Cell cycle distribution analysis was performed in cells after 48 h of exposure to batimastat, using PI solution with RNAse (Immunostep, Salamanca, Spain) according to manufacturer instructions. In brief, 1 × 10^6^ cells of each study condition were collected and washed with PBS for 5 min at 400 g. The pellet was resuspended in 200 µL of 70% ethanol solution with vortex agitation and incubated for 30 min at 4 °C. Then, cells were washed with PBS and resuspended in 300 µL of PI/RNase solution (Immunostep) for 15 min. Cells were analyzed on a FACSCalibur flow cytometer (Becton Dickinson), and the cell cycle distribution was analyzed using ModFit^LT^ Software v. 2.0 (Verity Software House, Topsham, ME, USA). The results were expressed as the percentage of cells in each cycle phase (G0/G1, S, G2/M). When present, a sub-G1 population was also identified and corresponded to apoptotic cells. Results represent the mean ± SEM of 3 independent experiments.

### 4.7. Gelatin Zymography Assay

Since the MMP-2 and -9 are gelatinases, it is possible to assess their gelatinolytic activity recurring to a gelatin zymography assay [33]. Cells were incubated with 5 µM and 7.5 µM BB-94 for 48 h in a serum-free conditioned media. After incubation, supernatants were collected and centrifuged (200× *g*, 5 min) to remove cellular remains. Then, 10 µL from each sample, derived from 5 × 10^6^ cells, were applied on a 10% polyacrylamide SDS-PAGE gel with 0.1% (*w*/*v*) gelatin, and electrophoresis was performed under nonreducing conditions. The gel was washed twice for 30 min, with constant shaking, in a renaturation buffer with 50 mM Tris-HCl (pH 7.6), 5 mM CaCl_2_, 150 mM NaCl, and 0.25% (*v*/*v*) Triton X-100 to remove SDS and renature the proteins. Then, it was incubated with the digestion buffer (50 mM Tris-HCl (pH 7.6), 5 mM CaCl_2_, 150 mM NaCl, and 0.1% (*v*/*v*) Triton X-100) for 36 h at 37 °C, and after that, gel was stained with 0.25% (*w*/*v*) Comassie Brilliant Blue in 50% (*v*/*v*) methanol and 10% (*v*/*v*) acetic acid. Enzymatic activity was detected as a white band on the resulting blue background of undigested gelatin. The intensity of the bands was measured using the Image Lab^®^ program v. 6.1 (BioRad, Hercules, CA, EUA). Results were normalized to control and represent the mean ± SEM of 3 independent experiments.

### 4.8. Western Blot Analysis

The activation of ERK and AKT pathways by BB-94 was determined by the p-ERK 1/2 and p-AKT expression on cell lines, according to Balça-silva et al. (2015) [34]. After incubation with 5 µM of BB-94 during 48 h, cells were collected and lysis buffer (RIPA buffer: 50 mM Tris-HCl (pH 8.0), 1% Nonidet P-40, 150 mM NaCl, 0.5% sodium deoxycholate, 0.1% SDS, 2 mM EDTA, and 1 mM DTT, freshly supplemented with protease and phosphatase inhibitor cocktails) was added at the end of incubation period. The nuclei and the insoluble cell debris were removed by centrifugation at 4 °C, at 12,000× *g* for 10 min. The postnuclear extracts were collected and used as total cell lysates. Protein quantification was carried out using the Pierce BCA Protein Assay Kit (ThermoFisher Scientific, Waltham, MA, USA), and the cell lysates were denatured at 95 °C for 5 min in sample buffer (0.125 mM Tris pH 6.8; 2% *w*/*v* SDS; 100 mM DTT; 10% glycerol and bromophenol blue) for its use in Western blot analysis. An amount of 30 μg of total protein was applied to each lane of a 12% sodium dodecyl–sulfate polyacrylamide gel electrophoresis (SDS-PAGE) to separate the proteins, which were then transferred to a PVDF membrane. Next, the membranes were blocked with 5% non-fat milk in Tris-buffered saline with 0.1% Tween-20 (TBS-T) for 1 h, incubated with specific primary antibodies overnight at 4 °C, washed with TBS-T, and incubated with peroxidase-conjugated antibodies. The immunodetection of p-ERK 1/2 (1:1000, Cell Signaling) and p-AKT (1:1000, Cell Signaling) expression was performed using the ECF method. Finally, the protein expression was quantified using the software Image Quant^TL^ for Windows (version 2005; Amersham Biosciences, Buckinghamshire, UK) with the expression of total ERK1/2 and total AKT as a loading control for p-ERK1/2, p-AKT, respectively. Results were normalized to control conditions and expressed in mean ± SEM of 3 independent experiments.

### 4.9. Statistical Analyses

Statistical analyses were performed with GraphPad Prism software, version 7.00 for Windows (GraphPad Software, v. 7.00 for Windows, San Diego, CA, USA). The IC_50_ determination was performed by non-linear dose–response curve fitting analysis. Student’s *t*-test, analysis of variance, Dunnett’s test, and Tukey’s test were used to compare the different groups. In this study, a significance level of *p*-value < 0.05 was considered. All values were expressed as mean ± SEM of indicated independent experiences.

## 5. Conclusions

Our results highlight the potential therapeutic effect of batimastat on hematological malignancies, particularly when administered daily to reduce toxicity.

## Figures and Tables

**Table 1:** Effects of BB-94 on cell cycle distribution.

	Sub-G_1_ (%)	G_0_/G_1_ (%)	S (%)	G_2_/M (%)
HL-60 Cells				
Control	2.7 ± 0.7	43.0 ± 1.0	47.0 ± 1.5	10.0 ± 2.5
BB-94 5 μM	14.5 ± 2.0	53.5 ± 0.6 **	36.0 ± 1.2	10.0 ± 0.6
BB-94 7.5 μM	17.7 ± 3.5	53.0 ± 0.6 **	40.0 ± 1.5	7.0 ± 1.2
NB-4 Cells				
Control	0.0 ± 0.0	53.3 ± 1.3	36.7 ± 1.8	10.0 ± 3.1
BB-94 5 μM	7.0 ± 1.2 **	48.3 ± 0.9 *	49.0 ± 1.0 ***	2.7 ± 1.8
BB-94 7.5 μM	7.7 ± 1.2 **	44.3 ± 1.2 **	51.3 ± 0.9 ***	4.3 ± 0.3
F36-P Cells				
Control	0.3 ± 0.3	55.8 ± 1.6	37.0 ± 2.2	7.3 ± 1.7
BB-94 5 μM	3.5 ± 0.9	53.8 ± 2.8	36.0 ± 3.0	10.3 ± 1.3
BB-94 7.5 μM	5.3 ± 0.3	53.0 ± 2.4	34.8 ± 2.5	12.0 ± 0.9
H929 Cells				
Control	1.8 ± 1.4	46.0 ± 1.2	40.3 ± 0.9	13.8 ± 2.1
BB-94 5 μM	5.5 ± 1.6	49.0 ± 0.8	37.3 ± 0.5	13.8 ± 1.3
BB-94 7.5 μM	9.5 ± 2.1 *	50.0 ± 0.8	37.8 ± 0.6	11.8 ± 0.9

Results represent mean ± SEM. * *p* < 0.05, ** *p* < 0.01, *** *p* < 0.001 compared with the control.

## Data Availability

All data generated or analyzed during this study are included in this published article.

## Supplementary Materials

The following supporting information can be downloaded at https://www.mdpi.com/article/10.3390/ijms25084554/s1.
