# Supplementary material for: Batimastat Induces Cytotoxic and Cytostatic Effects in In Vitro Models of Hematological Tumors
Source: Int J Mol Sci. 2024 Apr 22;25(8):4554. doi: 10.3390/ijms25084554 (PMC11050270; doi:10.3390/ijms25084554)
Supplement: Supplementary file 1 [file ijms-25-04554-s001.zip › ijms-2966782-supplementary.pdf]

Supplementary Material

Batimastat induces cytotoxic and cytostatic effects in in vitro models of hematological tumors

Raquel Alves [1,2,3,†], Ana Pires [1,4,†], Joana Jorge [1,2,3], Joana Balça-Silva [1,5], Ana Cristina Gonçalves [1,2,3,*], and Ana Bela Sarmento-Ribeiro [1,2,3,6]

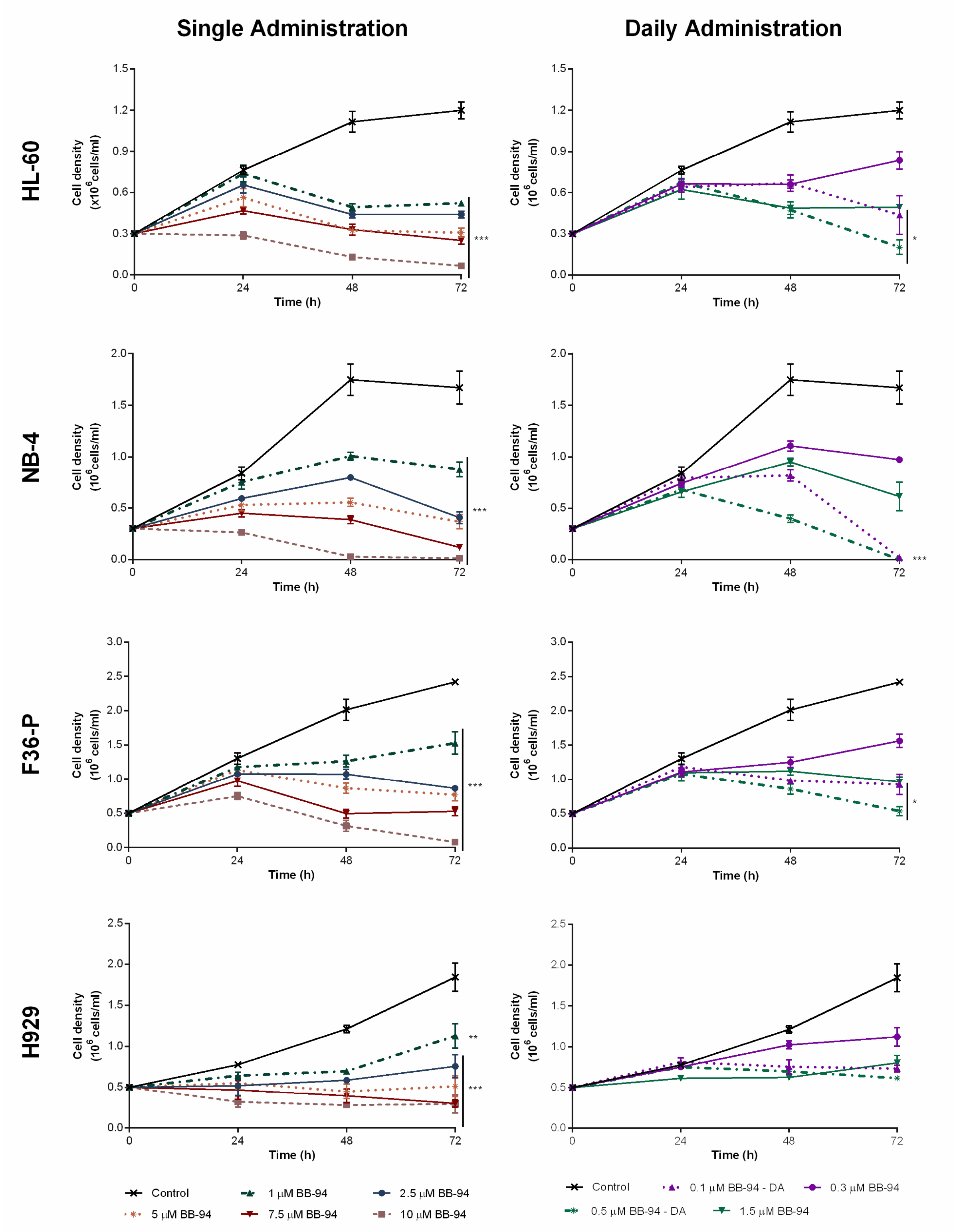

Supplementary Figure S1. Cell density curves of batimastat (BB-94) in a single administration and daily administration schedules for the four hematological cell lines. Cells were incubated in the absence and presence of different concentrations of BB-94 during 72h with different administration schemes. Results were expressed in number of cells (10^6 cell per mL) and represent the mean ± SEM obtained from 4 independent experiments. * $p<0.05$; ** $p<0.01$; *** $p<0.001$ (comparison with control).

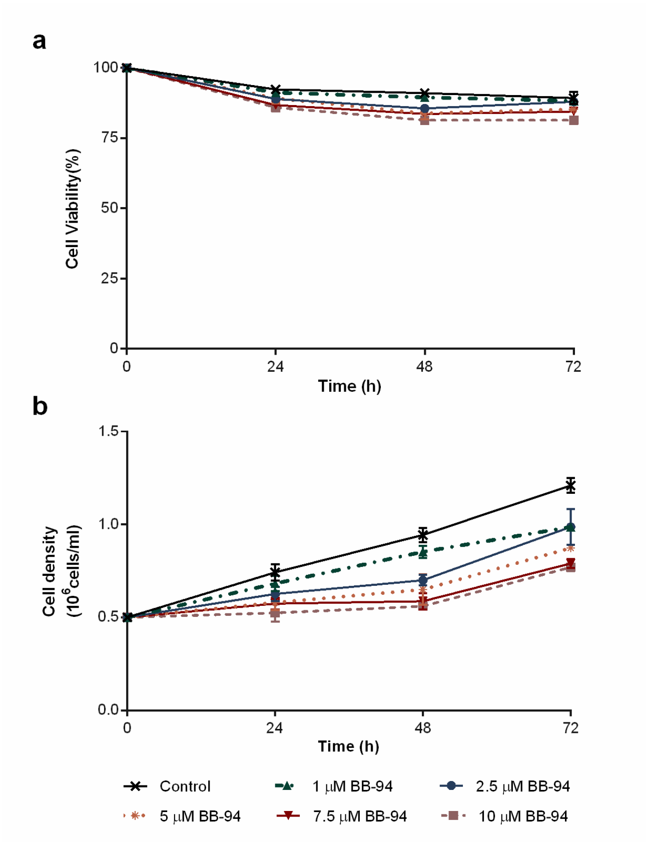

Supplementary Figure S2. Dose-response curves **(a)** and cell density curves (**b**) of batimastat (BB-94) in a single administration in IMC cell lines. Cells were incubated in the absence and presence of different concentrations of BB-94 during 72h. Results were expressed in percentage (%) and in number of cells (10^6 cell per mL), and represent the mean ± SEM obtained from 4 independent experiments.

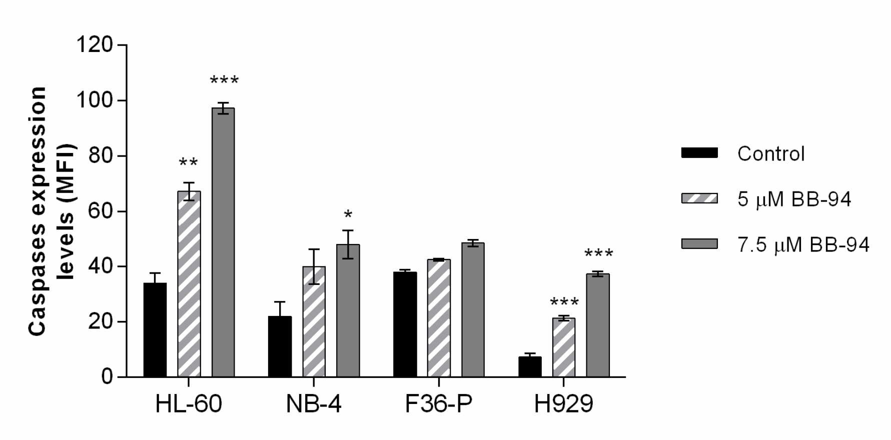

Supplementary Figure S3. Caspases expression levels induced by Batimastat. The caspases levels assessment was determined by flow cytometry using ApoSTAT probe as markers of apoptosis. Data are expressed d by mean fluorescence intensity (MFI) for ApoSTAT labeling Results were obtained after 48h of incubation and represent mean ± SEM of at least 3 independent experiments. * $p<0.05$; ** $p<0.01$; *** $p<0.001$ (comparison with control).

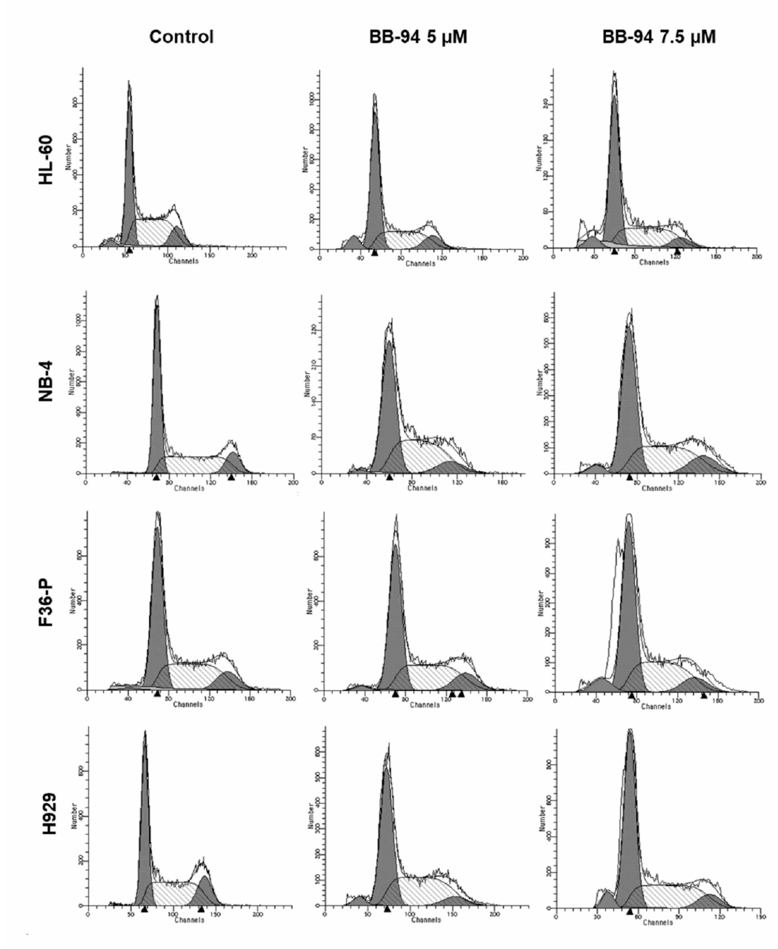

Supplementary Figure S4. Representative histograms of cell cycle distribution upon BB-94 treatment

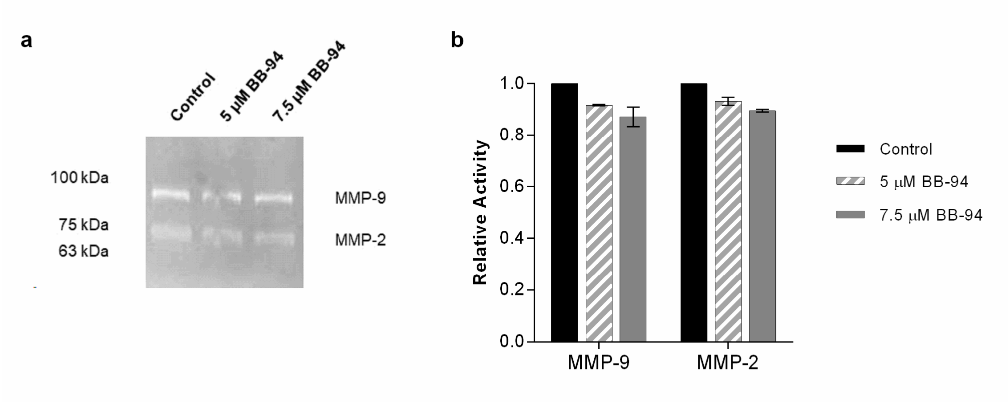

Supplementary Figure S5. Analysis of the effect of BB-94 on gelatinase secretion and activity in NB-4 cells. (**a**) A representative gel of gelatin zymography of untreated and BB-94-treated cells. The two bands observed correspond to both gelatinases, the MMP-2 with 72 kDa and the MMP-9 with 92 kDa. (**b**) The MMP-2 and MMP-9 activity were quantified and compared with the control. Data are represented as mean ± SEM of 3 independent experiments.
